# Unwinding JAZ7 – enigma to harmony

**DOI:** 10.1093/jxb/erw191

**Published:** 2016-05-28

**Authors:** Christine Shyu

**Affiliations:** Donald Danforth Plant Science Center, Saint Louis, MO 63132, USA

**Keywords:** Arabidopsis, COI1, fungal pathogen, JAZ, JAZ7, jasmonate, senescence.

**JASMONATE ZIM-DOMAIN (JAZ) proteins are primary transcriptional repressors in the jasmonate (JA) signaling pathway that regulate a broad range of JA-dependent responses. A mechanistic mode of action is well established, but what are the biological roles of individual JAZs, and how do they contribute to the specification of JA signaling outputs? Two recent articles in *Journal of Experimental Botany* reveal roles of *JAZ7* in regulating dark-induced senescence and susceptibility to fungal pathogens.**

Jasmonates (JAs) regulate a broad range of processes, from growth and development to biotic and abiotic stress responses. JASMONATE ZIM-DOMAIN (JAZ) proteins are key regulators in controlling JA signaling outputs. Since the discovery of *JAZ*s in 2007, over 100 research articles have been published on the characterization of JAZ functions, genomic or transcriptomic analyses of *JAZ*s, or the identification of down-stream transcription factors in the JA signaling pathway through yeast two-hybrid screens using JAZs as bait (reviewed in Pauwels and Goossens, 2011; Shyu and Brutnell, 2015). Biochemical and molecular functions of JAZ proteins have been extensively studied, but surprisingly little is known about the biological role of JAZs and how they contribute to specific JA signaling outputs.

## Enigmatic JAZ7

JAZs were identified in 2007 as the missing link between the F-box protein CORONATINE INSENSITIVE 1 (COI1) and the transcription factor MYC2, which mediates JA responses (Chini *et al.*, 2007; Thines *et al.*, 2007; Yan *et al.*, 2007). JAZ proteins function as transcriptional repressors that repress gene expression by bridging co-repressors NOVEL INTERACTOR OF JAZ (NINJA)/TOPLESS and transcription factors to repress JA-responsive gene expression. Upon accumulation of bioactive JA, JAZs form a co-receptor complex with COI1 to perceive JA. This interaction leads to ubiquitination and degradation of JAZ proteins, resulting in activation of JAZ-interacting transcription factors and JA-responsive gene expression (reviewed in Pauwels and Goossens, 2011).

Among the 13 JAZ proteins in Arabidopsis, JAZ7 is one of the more enigmatic members of the family. Unlike most JAZs, JAZ7 does not form homo or heterodimers with other JAZ members, and interacts with very few transcription factors, namely MYC3 and MYC4 (Chung and Howe, 2009; Fernandez-Calvo *et al.*, 2011; Thatcher *et al.*, 2016). JAZ7’s closest homolog is JAZ8, which shares an N-terminal ERF-associated amphiphilic repression (EAR) domain and was characterized to be a stable transcriptional repressor even in the presence of JA (Shyu *et al.*, 2012). Similar to JAZ8, JAZ7 does not interact with the co-repressor NINJA (Pauwels *et al.*, 2010). It was speculated that JAZ7 would have similar molecular functions as JAZ8, but no biochemical or genetic characterization was reported.

In Yu *et al.* (2016), it is shown that *jaz7* has a loss-of-function phenotype – something that has only been detected with *jaz9* and *jaz10* loss-of-function alleles and not with the other 10 *JAZ* genes to date (Cerrudo *et al.*, 2012; Demianski *et al.*, 2012; Yang *et al.*, 2012; Leone *et al.*, 2014; Yu *et al.*, 2016). Working independently, Thatcher *et al.* (2016) characterized the same T-DNA insertion allele, which they named *jaz7-1*, along with an activation-tagged allele *jaz7-1D*. These studies mutually reveal the biological function of *JAZ7* in Arabidopsis.

## Dark-induced senescence

Using detached leaves, Yu *et al.* (2016) showed that transcripts from *JAZ7* are rapidly induced in the dark, along with several other *JAZ* genes. Screening of multiple *jaz* T-DNA insertion lines then demonstrated that the loss-of-function *jaz7* allele was more sensitive to dark-induced senescence, with enhanced leaf yellowing and reduced chlorophyll content compared to wild type. This phenotype was complemented by *35S::JAZ7* in the *jaz7* background. Overexpression of *JAZ7* in the wild-type background showed no significant senescence phenotype, but had reduced H_2_O_2_ content compared to wild-type plants. These results collectively suggest that *JAZ7* plays a role in inhibiting dark-induced senescence. Genetic analysis of *jaz7* crossed with *coi1* or *myc2* indicated that *JAZ7*-regulated senescence is dependent on *COI1* and the regulation is likely through *MYC2*. A microarray analysis was then performed in WT and *jaz7* in both control and dark conditions to identify genes regulated by *JAZ7* in response to dark. The authors concluded that JAZ7 plays a role in inhibiting dark-induced senescence by controlling senescence, cell death, and defense-responsive gene expression.

## Fungal resistance

Thatcher *et al.* (2016) found *JAZ7* in a very different way. Using the agriculturally destructive fungal pathogen *Fusarium oxysporum*, *jaz* T-DNA insertion alleles were screened for altered susceptibility to *F. oxysporum*. The *jaz7-1D* line was identified among other alleles to be more susceptible to *F. oxysporum* treatment. Interestingly, the T-DNA insertion in *jaz7-1D* was in the promoter region of *JAZ7* and led to increased expression of *JAZ7*, mimicking a *JAZ7* overexpression line. In contrast to the *JAZ8* overexpression line and other dominant-negative *JAZ* lines that are less sensitive to JA (Thines *et al.*, 2007; Chung and Howe, 2009; Shyu *et al.*, 2012), *jaz7-1D* showed hypersensitive phenotypes to JA including increased JA-induced root growth inhibition and increased JA-responsive gene expression in response to stress in addition to increased susceptibility to *F. oxysporum*. These results suggest that *jaz7-1D* either functions as a dominant-negative allele, or that *JAZ7* plays a positive role in promoting JA responses, which is contradictory to biochemical characterization of JAZs published to date. Even more puzzling, transgenic lines overexpressing *JAZ7* did not reproduce phenotypes observed in *jaz7-1D*. With careful genetic characterization of *jaz7-1D* along with sequencing of the *JAZ7* transcripts in both WT and *jaz7-1D* and analysis of RNAseq data from Yan *et al.* (2014), the authors ruled out possibilities of additional T-DNA insertions, mutations in the *JAZ7* transcript, or alternative splice forms causing the *jaz7-1D* phenotype.

To clarify the molecular function of JAZ7, Thatcher *et al.* conducted a series of biochemical and molecular studies and concluded that JAZ7 is indeed a transcriptional repressor. JAZ7 interacted with co-repressor TOPLESS likely through its N-terminus EAR motif, and repressed target gene expression in transient transcriptional activity assays in an EAR-dependent manner. Though these studies support the hypothesis of JAZ7 functioning similarly to the stable transcriptional repressor JAZ8, the *jaz7-1D* hypersensitive phenotype is still hard to explain. The authors speculate that *jaz7-1D* phenotypes may be due to ectopic cell- or tissue-specific expression caused by the T-DNA insertion, or that *jaz7-1D* may have hypersensitive phenotypes due to sequestering transcriptional repressors such as JAM1 (Nakata *et al.*, 2013). Interestingly, microarray and qRT-PCR analyses of *jaz7-1D* showed that expression of genes involved in senescence such as *SAG12* and *DIN11* were induced in *jaz7-1D* and that the *jaz7-1* loss of function allele exhibited wild-type *Fusarium*-induced senescence responses. This seems contradictory to the observation of *JAZ7* negatively regulating senescence as reported by Yu and colleagues. Taken together, it is hard to rationalize the overexpression and *jaz7-1D* phenotypes with the loss-of-function allele.

## JAZ harmony – who does what?

Though biochemical and molecular functions of JAZ proteins are fairly conserved within the JAZ family, it has become clear that specific JAZs contribute to different biological functions that JA regulates (Box 1). Mutants in *jaz7* are more responsive to dark-induced leaf senescence while *jaz6* mutants are less sensitive (Yu *et al.*, 2016). The *jaz9* mutant is late flowering (Yang *et al.*, 2012) and *jaz13*/*jaz7*/*jaz8*/*jaz10* mutants are hypersensitive to JA-induced root growth inhibition (Thireault *et al.*, 2015). In addition to loss-of-function phenotypes, divergent biological functions of each JAZ have been proposed based on JAZ-interacting transcription factors that regulate specific JA responses. For example, yeast two-hybrid screens using JAZ1 as bait led to the identification of AP2 transcription factors – TARGET OF EAT 1 (TOE1) and TOE2 – that target *FLOWERING LOCUS T* to delay flowering time (Zhai *et al.*, 2015). Among all JAZs tested, TOE1 and TOE2 only interact with JAZ1, JAZ3, JAZ4 and JAZ9, suggesting that these specific JAZs are involved in JA-mediated flowering time regulation. These interaction-based discoveries are informative, but it is worth mentioning that most of these interactions are deduced from heterologous systems, and phenotypic characterizations are generally performed on alleles that have altered expression of down-stream transcription factors.

**Box 1. Model for JAZ specification of diverse JA-mediated responses**

Lines are drawn based on phenotypes reported in loss-of-function, overexpression, or dominant-negative lines of the JAZ proteins indicated. JAZX represents JAZs other than those specified; dashed lines indicate speculative interactions.

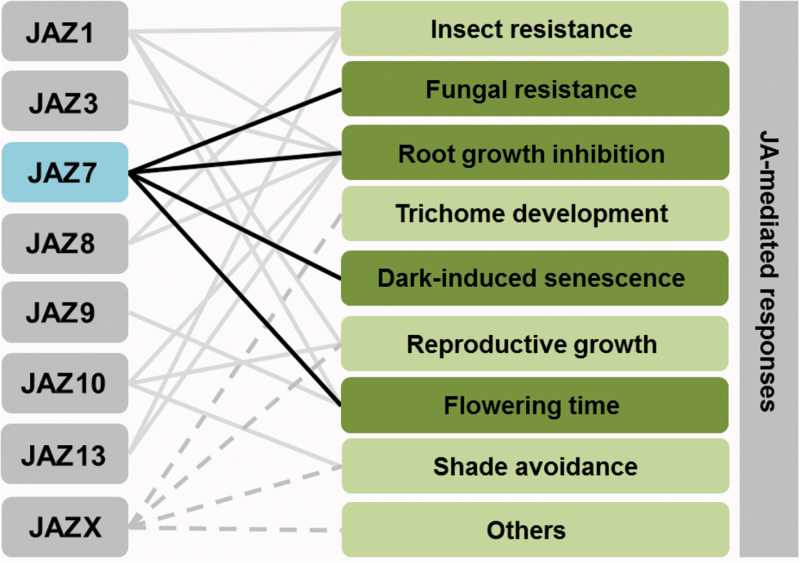

Most *JAZ* overexpression lines have no phenotypes, presumably due to their instability in the presence of bioactive JA. This is with the exception of *JAZ8* and *JAZ13*, where overexpression of *JAZ8* and *JAZ13* leads to decreased sensitivity to JA-induced root growth inhibition and increased susceptibility to insect feeding (Shyu *et al.*, 2012; Thireault *et al.*, 2015). Overexpression of alternative splice forms of *JAZ10* that produce a stabilized *JAZ10* variant also leads to male sterility in addition to decreased sensitivity to JA-induced root growth inhibition (Chung and Howe, 2009). Though *jaz7-1D* is hypersensitive to JA-induced root growth inhibition, it is interesting that both *jaz7-1D* and *JAZ9* overexpression lines are early flowering (Thatcher *et al.*, 2016; Yang *et al.*, 2012). This suggests overlapping functions for *JAZ7* and *JAZ9* to promote flowering.

## Future perspectives

We are just starting to get a glimpse of the biological function of JAZ proteins in Arabidopsis. Characterization of *jaz* mutants needs to be more comprehensive and detailed to get a broader view of JAZ function. JAZ proteins are grouped into different clades based on sequence similarity (Chico *et al.*, 2008), and thus higher order mutants are also desirable given the high degree of redundancy among *JAZ*s (Thireault *et al.*, 2015). The contradicting phenotypes of the *jaz7-1D* mutant also suggest that the COI-JAZ-TF signaling pathway may be more complex. Therefore biochemical approaches to identify JAZ complexes *in vivo* are needed to provide more pieces of the JA puzzle. In addition to Arabidopsis *JAZ*s, there is a much more diverse pool of *JAZ* genes in monocots, particularly grasses. JA mutants in rice and maize also reveal unique roles for JA in regulating spikelet formation, stem elongation and sex determination (reviewed in Shyu and Brutnell, 2015). Given the rapid development of CRISPR technology and other genome editing tools (reviewed in Liu *et al.*, 2016), it will become increasingly straightforward to manipulate *JAZ* sequences to study the biological function of JAZs in different species. This will help us decipher the complex network of JA biology in plants, and provide possibilities to fine-tune JA responses in economically important crop species.
